# Diagnosis and Monitoring Pathways Using Non‐Invasive Tests in Patients With Alpha‐1 Antitrypsin Deficiency‐Associated Liver Disease: Results From an Expert Delphi Panel

**DOI:** 10.1002/ueg2.70009

**Published:** 2025-03-12

**Authors:** Virginia C. Clark, Mark A. Price, Jon Russo, Rohit Loomba, Alice M. Turner, Pavel Strnad

**Affiliations:** ^1^ University of Florida Gainesville Florida USA; ^2^ RTI Health Solutions Research Triangle Park North Carolina USA; ^3^ University of California San Diego School of Medicine San Diego California USA; ^4^ University of Birmingham Birmingham UK; ^5^ University Hospital RWTH Aachen Aachen Germany

**Keywords:** alpha‐1 antitrypsin deficiency, Delphi, liver fibrosis

## Abstract

**Background and Aims:**

The severe alpha‐1 antitrypsin deficiency (AATD) genotype Pi*ZZ increases the risk of liver disease (AATD‐LD) and lung disease. While non‐invasive tests (NITs) are widely used for fibrosis stage and monitoring of all liver diseases, the consensus for use in AATD‐LD is limited. A Delphi panel study was conducted to address this need.

**Method:**

Healthcare providers who managed at least two patients with AATD‐LD in the past two years participated. Two iterative surveys were developed and administered. The second survey clarified the results from the first and provided deeper feedback. As follow‐up, a real‐time consensus‐building exercise focused on survey topics without consensus. Controlled feedback was anonymous.

**Results:**

A total of 20 AATD experts (hepatology [*n* = 9], pulmonology [*n* = 6], transplant hepatology [*n* = 3], gastroenterology [*n* = 1], and hepatology and transplant hepatology [*n* = 1]) completed the study. A strong consensus was achieved around the use and evaluation of NITs for risk stratification and monitoring. All panelists agreed that vibration‐controlled transient elastography (VCTE) is the preferred NIT for the initial assessment of AATD‐LD owing to its accessibility and reliability. Magnetic resonance elastography and enhanced liver fibrosis tests were also considered valuable. Most (85%) agreed that VCTE < 8 kPa could indicate no or mild fibrosis and VCTE ≥ 8 kPa could indicate clinically significant fibrosis, which may correspond to fibrosis stage ≥ F2 on the METAVIR scale. Most (85%) agreed that VCTE ≥ 13 kPa may indicate cirrhosis.

**Conclusion:**

Utilizing the Delphi technique, this study identified a clinically applicable framework for the diagnosis and monitoring of AATD‐LD. A high level of agreement emerged regarding preferred NITs and their usage, risk stratification and monitoring in the context of AATD‐LD management. The results provide a foundation for future efforts into NIT validation and the development of clinical guidelines for AATD‐LD.

## Graphical abstract

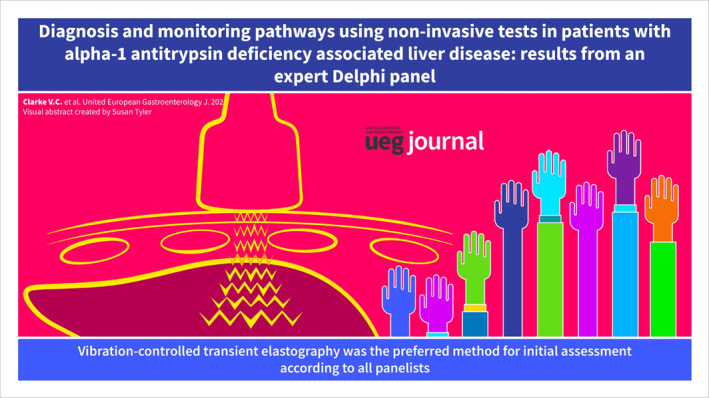

1

**Summary**

- Established knowledge on this subject
  ◦ Severe alpha‐1 antitrypsin deficiency (AATD) is caused predominantly by homozygous Pi*Z mutation (Pi*ZZ genotype).
  ◦ Pi*ZZ subjects are highly predisposed to the development of AATD‐associated liver disease (AATD‐LD), but no detailed guidelines for liver monitoring exist.
- Significant and/or new findings of this study?
  ◦ A Delphi panel study was conducted to reach a consensus for the use of non‐invasive tests (NITs) in AATD‐LD.
  ◦ Vibration‐controlled transient elastography (VCTE) emerged as the preferred NIT for the initial assessment.
  ◦ Most panelists agreed that VCTE cut‐offs should be used for risk stratification and intermittent monitoring for progression.

**Abbreviations**
AAT: alpha‐1 antitrypsin
AATD: alpha‐1 antitrypsin deficiency
AATD‐LD: AATD‐associated liver disease
APRI: aspartate aminotransferase‐to‐platelet‐ratio index
COPD: chronic obstructive pulmonary disease
ELF test: enhanced liver fibrosis test
F: fibrosis stage
FIB: fibrosis 4 index
LSM: liver stiffness measurement
METAVIR: meta‐analysis of histological data in viral hepatitis
MRE: magnetic resonance elastography
NIT: non‐invasive test
RTI‐HS: RTI Health Solutions
VCTE: vibration‐controlled transient elastography

## 2 Introduction

Alpha‐1 antitrypsin deficiency (AATD) is a genetic disorder resulting from mutation in alpha‐1 antitrypsin (AAT) gene affecting the production or secretion of the protein [1, 2]. AAT is a major protease inhibitor normally present in the bloodstream and the lungs. Its diminished levels lead to unopposed proteolytic damage in the lungs and development of emphysema and/or chronic obstructive pulmonary disease (COPD). A build‐up of abnormal AAT in the liver predisposes to liver injury and fibrosis, and the accumulation can be seen in the form of globules that are periodic acid diastase positive (PAS+D) [3]. While more than 150 AAT mutations have been described, homozygous Glu342Lys substitution (rs28929474), termed the Pi*ZZ genotype, is responsible for the vast majority of severe AATD cases [1, 2, 3, 4]. Although this genetic condition confers a markedly increased risk for both organs, the lung and liver phenotype of Pi*ZZ subjects is highly variable. Some patients remain asymptomatic, while others experience early symptoms, including shortness of breath with mild activity, reduced ability to exercise, wheezing, and fatigue [5]. Lung disease constitutes the leading cause of Pi*ZZ‐related mortality. Multiple guidelines offer recommendations for clinical surveillance and treatment of AATD‐related lung disease, including standard COPD therapies as well as augmentation therapy and a weekly intravenous application of purified human AAT [4, 5, 6].

Lung and liver diseases develop independently of each other, and both of them constitute an important cause of mortality in AATD [7, 8]. Monitoring for the development of liver disease may be done across multiple specialties, and current recommendations focus on liver enzyme monitoring, and liver imaging with ultrasound (Table 1), which is not sensitive to detect liver disease [5, 9, 10]. Liver biopsy offers a complete evaluation of AATD‐LD since it quantifies both the amount of AAT by PAS+D globules and liver fibrosis stage primarily assessed on the meta‐analysis of histological data in viral hepatitis (METAVIR) scale [7, 11, 12]. Fibrosis stage constitutes an established, etiology independent surrogate of liver‐related mortality [12]. In clinical practice, the use of noninvasive tests (NITs) has largely replaced routine liver biopsy and provides an estimate of fibrosis stage [13, 14]. Imaging‐based markers include liver stiffness measurement (LSM) via vibration‐controlled transient elastography (VCTE) and magnetic resonance elastography (MRE) [15, 16]. Blood based NITs include aspartate aminotransferase‐to‐platelet‐ratio index (APRI) and fibrosis‐4 index (FIB‐4) that rely on routinely available test [16, 17]. Finally, several surrogates, such as the pro‐C3 or enhanced liver fibrosis test (ELF test), directly assess the production/turnover of extracellular matrix that constitutes the structural basis of fibrosis. The evidence for the diagnostic accuracy of NITs in AATD‐LD is limited to a few studies with direct comparison to liver biopsies [7, 18, 19]. For the first time, a recent clinical practice guideline recommended LSM use in Pi*ZZ adults to estimate fibrosis, but it did not offer any guidance for a threshold for risk stratification or the role of repeat monitoring with NITs [20]. No accepted thresholds for the use of LSM in AATD‐LD for risk stratification exist, but ≥ 7.1 kPa for fibrosis stage ≥ 2 and ≥ 10 kPa for fibrosis stage ≥ 3 have been reported [8]. To address the current situation, we conducted a Delphi panel exercise to (1) elicit information on how NITs are used in clinical practice for AATD‐LD (2) develop a consensus with physicians across disciplines on the usefulness and thresholds of various noninvasive tools and (3) develop an assessment pathway useful in clinical care of adult Pi*ZZ patients.

**TABLE 1:** Current recommendations on diagnosis, monitoring and fibrosis staging in AATD‐LD.

	Organization	Recommendations/Statements
Diagnostic testing AATD‐LD	Alpha‐1 foundation	All individuals with unexplained chronic liver disease should be tested for AATD
	ATS/ERS	Individuals with unexplained liver disease, including neonates, children, and adults, particularly the elderly should be tested for AATD
Follow up AATD‐LD	Alpha‐1 foundation	Monitoring for liver disease at annual intervals (or more frequently as indicated by clinical circumstances) with a focused exam for signs of liver disease, liver ultrasound, and monitoring of AST, ALT, GGT, albumin, bilirubin, INR, and platelets is recommended
	ATS/ERS	In the absence of firm evidence, clinical management should include regular assessment of liver function test and ultrasound examination
	EASL	Liver stiffness measurements can be used in adults with Pi^*^ZZ to estimate the level of histological fibrosis
		Liver biopsy should be considered when careful non‐invasive evaluation remains inconclusive
		Lifestyle counseling is recommended as smoking, obesity, and alcohol consumption have negative effects of the health of patients with AATD
Initial fibrosis staging^a^	EASL	Non‐invasive scores, serum markers, liver stiffness, and imaging methods can identify advanced fibrosis in patients at risk from low prevalence populations better than clinical acumen alone
	AASLD	Recognizing that liver histology is an imperfect reference standard, prior to considering a liver biopsy to assess fibrosis staging in patients with CLD, AASLD recommends using blood and imaging‐based NILDA as the initial tests to detect significant (F2‐4) to advanced fibrosis (F3‐4) and cirrhosis (F4)
	NICE	Offer transient elastography to diagnose cirrhosis for HCV, alcohol related liver disease, and MASLD with advanced fibrosis by ELF test

^a^ No studies specific to AATD were included in evidence for EASL or AASLD clinical practice guidelines or NICE recommendations.

## 3 Methods

### 3.1 Study Design

The Delphi panel collected information to glean insights, feedback, and suggestions regarding the monitoring and noninvasive assessment of adult patients with AATD‐LD associated with the Pi*ZZ genotype. The Delphi panel exercise explored how patients are monitored across the specialties that care for AATD such as hepatology, gastroenterology, and pulmonology. The steps of the Delphi panel are shown in Supporting Information S1: Figure S1. A brief study description was submitted on 21 April 2023 to RTI International's institutional review board under application number STUDY00022452. The study was granted an exemption from review and received a determination of not human subject's research. VCC, AT, RL, and PS constituted the scientific advisors for the study and had oversight of study design and development of study materials. Additional roles included recommendations for panelists and ensuring that the study scope and key questions addressed were appropriate. The advisors reviewed the results after each phase of data collection to ensure that quality information was gathered and assist in interpretation of the findings. However, they were restricted from making alterations to data collected from the panelists and presenting their personal opinions.

### 3.2 Study Setting and Study Population

The study was conducted virtually with clinicians from selected countries to capture practice patterns across the United States, Canada, the United Kingdom, and the European Union. To conduct the study, RTI Health Solutions (RTI‐HS) received a funding from Takeda Pharmaceuticals. RTI‐HS contacted the panelists and compensated them for their time. For this, they identified the authors of relevant AATD‐LD studies and incorporated suggestions from the study scientific advisors. Delphi panelists met the following criteria (based on self‐report): (i) Serve as an expert in hepatology, gastroenterology, or pulmonology; (ii) Follow at least 2 patients with AATD in the last 24 months; (iii) Ability to provide an informed consent. Using a snowballing recruitment technique (using the professional network of newly recruited panelists who then nominated colleagues who would also be eligible), 21* practicing clinicians who are experts in AATD‐LD were recruited. Snowball recruitment is a method to identify potential experts to participate in a study, especially when expertise is rare, such as AATD‐LD [18]. The engagement with clinicians was single‐blinded, meaning panelists knew the identity of the study sponsor, but the sponsor remained blinded to clinician identities.

### 3.3 Data Collection Schedules and Procedures

The data collection schedule is presented in Supporting Information S1: Table 1. Delphi questionnaires were developed and finalized by the RTI‐HS study team, each requiring approximately 30 min to complete. The specific questionnaires used are presented in the supplemental material as questionnaires 1 and 2. Qualtrics, an online data collection platform, was utilized to program and administer the questionnaires. Seminal papers were distributed approximately one week prior to the first questionnaire and served as a foundation for all Delphi panelists. The second questionnaire summarized and presented results from the initial questionnaire and posed follow‐up questions to elicit deeper feedback. In the second questionnaire, areas of agreement and disagreement and areas of priority were illuminated. Then, two virtual consensus‐building exercises were conducted in real time to accommodate the schedules of the panelists. Consensus‐building exercises addressed results from the questionnaires where areas of disagreement remained. When a lack of consensus was present, discussion occurred to make the necessary revisions required for endorsement. Panelists were blinded to one another to maintain anonymity, reduce bias, and encourage open participation. Key topics were summarized via thematic analysis, which entailed grouping all panelist responses under a given topic and then developing a comprehensive but aggregated summary of all learnings on that topic. An experienced focus group moderator from the RTI‐HS team led the 90‐min discussions, and additional team members with expertise in qualitative research were present to ensure adequate discussion probes were implemented; each topic was fully covered, and all results were reported and confirmed.

### 3.4 Data Analysis

This was a qualitative study. Participant responses were analyzed across the questionnaires. The consensus‐building exercise for areas of consensus and disagreement followed the Recommendations for the Conducting and Reporting of Delphi Studies checklist for the methodology and reporting of results [21]. For open‐ended questions, dominant trends were identified in each response and compared with the results of all responses to generate themes or patterns in the way panelists provided responses via constant comparative analysis [20]. We used standard qualitative data collection and analytical methods that follow two main guiding principles: researcher neutrality and systematic process. While formal coding is generally not undertaken in qualitative Delphi panel research, constant comparative analysis ensures that comments resulting from multiple questions but related to a single theme are grouped and summarized together. These summaries by theme are then presented back to panelists in subsequent rounds of data collection for additional revisions and ultimate endorsement. In cases where a panelist was unable to provide an endorsement, reasons were captured. Additionally, where appropriate, we report simple descriptive statistics. For levels of consensus among the 20 panelists, a rubric was employed to categorize agreement levels and to delineate degrees of agreement and acknowledge potential variations within the consensus statements developed over the course of the study: Complete consensus is defined by unanimous agreement, strong consensus by 80% to 99% agreement or 100% agreement with minor modifications and moderate consensus by 60% to 79% agreement, with or without modifications. Weak consensus refers to 50%–59% agreement and offers an opportunity for explanation due to regional or specialty differences or an opportunity to be explored in future studies.

## 4 Results

The Delphi process took approximately seven months (June 2023–December 2023). 35 potential panelists were invited to participate in the study, with several declining or not responding to multiple invitations. Characteristics of the panelists who completed the study are represented in Table 2. The composition of the panel reflects the perspective of clinicians in the UK, US, and Europe with breadth and depth of experience in the clinical care of individuals with Pi*ZZ AATD. The geographical distribution and specialties of individuals who declined to participate were comparable to the panelists; the reasons for their lack of participation are not known.

**TABLE 2:** Demographics for panelists (*n* = 20).

	*n*
Specialty	
Gastroenterology	1
Hepatology	9
Pulmonology	6
Transplant hepatology	3
Hepatology and transplant hepatology	1
Time in practice (post residency)	
0–5 years	1
6–10 years	5
11–15 years	4
16–20 years	3
> 20 years	7
Country	
Austria	1
Belgium	1
Canada	1
France	2
Italy	1
Sweden	1
United Kingdom	1
United States	12
Number of patients with AATD with Pi*ZZ genotype in the last 24 months	
Mean	32
Range	2 to 200
Total number of patients followed by all 20 participants	652
Current practice setting	
University‐based hospital, academic center, or clinic	17
Tertiary care center	2
Community hospital or clinic	1

### 4.1 Delphi Round 1

The first questionnaire was broad in scope and intended to elicit surface level information from the panelists regarding the approach to diagnostic tools used and how to monitor the progression of AATD‐LD. The results showed that multiple tools are used in combination for a clinical assessment of AATD‐LD, and VCTE emerged as the primary non‐invasive tool used in clinical practice. Interestingly, there was complete consensus around a 2–3 years monitoring interval for liver disease in the absence of additional risk factors. Practice setting emerged as an important determination of which NIT is used. The consensus statements and strength of consensus from the first survey are shown in Supporting Information S1: Table 2. No consensus was reached on appropriate society guidelines to use in clinical practice when evaluating individuals with AATD for liver disease. A summary of current guidance on diagnosis, monitoring and staging of AATD‐LD as well as use of NITs in liver disease is shown in Table 3.

### 4.2 Delphi Round 2

The second questionnaire results informed the development of the initial consensus statements pertaining to the diagnosis of AATD‐LD and the frequency of monitoring for progression. The results showed that a classification system for AATD‐LD as well as a stepwise approach to the evaluation of AATD‐LD is needed. Further insight showed that VCTE was recommended as the first step in fibrosis staging, and MRE is useful but limited by cost and availability. Complete consensus from the panel was present on the need for data on liver progression in AATD‐LD and the validity of NIT to monitor disease progression. Most importantly, the panel agreed that fibrosis stage was an important determinant of how often individuals with AATD‐LD should be evaluated. The strength of consensus from the second round was 100% for 6 of 8 statements as shown in Supporting Information S1: Table 3.

### 4.3 Consensus Building

After the first two rounds, there was a lack of consensus around important issues for the use of NITs in clinical practice. These included which METAVIR fibrosis score was most important for risk stratification, the corresponding NIT values for METAVIR stages, the ability of NITs to distinguish between F2 and F3, and use of multiple NITs in a multi‐staged approach for fibrosis progression monitoring. Uncertainty also remained around which guidelines were most relevant for clinical use in AATD‐LD. Consensus building exercises were structured around these areas of disagreement to explore the topics further. The results of the consensus building exercises, findings endorsed by the panelists, and representative quotes are shown in Supporting Information S1: Table 4. All data and live discussions were incorporated in the final consensus statements, which underwent only minor revision after the final input of the panelists.

**FIGURE 1:**
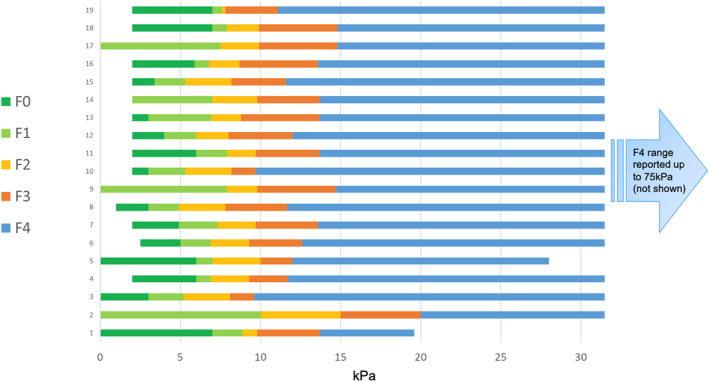
Vibration controlled transient elastography (VCTE) range values suggested by the Delphi panelists. Based on the suggested ranges of VCTE values for F0 and F1 fibrosis, a strong consensus was reached for a VCTE value of < 8 kPa to indicate no or mild fibrosis and VCTE values ≥ 8 kPa to indicate clinically significant fibrosis as ≥ F2. Most (85%) panelists agreed a threshold VCTE ≥ 13 kPa for cirrhosis; however, two panelists suggested a different value and one was unsure.

The final consensus statements shown in Table 3 were reviewed, revised, and endorsed by the Delphi panelists. These address the use of elastography, ultrasound, and liver biopsy in the evaluation and monitoring of individuals with AATD‐LD. Complete consensus was reached on the usefulness of VCTE, scope of initial evaluation of AATD‐LD, as well as on frequency of evaluation based on METAVIR score. The same level of agreement was also obtained for the use of liver biopsy to exclude competing causes of liver injury or for fibrosis staging in discordant cases as well as signs of advanced liver disease and triggers for liver transplant consideration. Based on suggested ranges of VCTE values for fibrosis, strong consensus emerged in support of the final proposed VCTE cutoff value of < 8 kPa to indicate no or mild fibrosis (Figure 1). The suggested thresholds for fibrosis staging are shown in Table 3. Two panelists proposed slightly different cutoffs for F3 and F4. One panelist did not support ≥ 8 kPa as a threshold that could distinguish between F1 and F2. While the use of MRE, FIB‐4, and APRI as NITs was explored by the panel to generate the initial consensus statement, the panel could not agree on thresholds corresponding to METAVIR fibrosis scores. As a result, MRE, FIB‐4, and APRI were not included in the final consensus statements or monitoring approach. In the early data collection, the use of ELF as an NIT for monitoring AATD‐LD patients was not thought to be helpful. During the consensus building stage, additional input from the panelists provided more contextualization for the usefulness of ELF alongside other NITs and clinical judgment.

**TABLE 3:** Final consensus statements.

Elastography
VCTE is the most used NIT when evaluating a patient with AATD‐LD for the first time (*complete consensus*) Consequently, elastography (i.e., VCTE, MRE) is the most valuable and widely available noninvasive monitoring approach to evaluate AATD‐LD progression (*strong consensus*) MRE as a diagnostic and/or monitoring approach is highly sensitive and specific for detection of advanced fibrosis. However, it is limited due to cost and lack of availability in some regions (*strong consensus*)
Ultrasound
Traditional ultrasound can be used in monitoring a patient with AATD‐LD (e.g., hepatocellular carcinoma screening, detection of steatosis, identification of nodular liver tissue, structural, or biliary disease, cirrhosis screening, and for monitoring of fibrosis progression in patients with BMI > 35). However, it has limited sensitivity for differentiating progression in early fibrosis. (strong consensus)
Initial evaluation
The most common clinical approach when evaluating a patient with AATD‐LD for the first time is referral to a hepatologist and use of laboratory measures, liver stiffness measurement (by elastography), and ultrasound. (complete consensus)
Frequency of liver monitoring
Frequency of evaluation of patients with AATD‐LD is based on METAVIR score. Lower METAVIR scores should be evaluated every 2–3 years and higher METAVIR scores should be evaluated every 6–12 months (complete consensus)
Use of biopsy
Liver biopsy may be considered to exclude competing causes of liver injury and chronic liver diseases or for fibrosis staging when results from two or more NITs for fibrosis are discordant (complete consensus)
Advanced disease and liver transplant
Clinical signs and symptoms of advanced liver disease include clinical evidence of portal hypertension (esophageal varices, splenomegaly, ascites), child‐pugh class B (score 7–9), hepatocellular carcinoma, an elevated MELD score > 15, evidence of liver dysfunction/deterioration including hepatic encephalopathy and sarcopenia all may indicate that a patient should be considered for liver transplant. (complete consensus)
AATD‐LD risk stratification
The most important risk stratification is the transition from F1 to F2 on the METAVIR scale. Differentiation of F0 versus F1 and F2 versus F3 is clinically less meaningful (strong consensus)
Risk stratification for AATD‐LD fibrosis
VCTE < 8 kPa may correspond to no or mild fibrosis (METAVIR score F0/F1) VCTE ≥ 8 kPa may correspond to clinically significant fibrosis (METAVIR score ≥ F2) VCTE ≥ 10 kPa may correspond to clinically significant fibrosis (METAVIR score ≥ F3) VCTE ≥ 13 kPa may correspond to possible cirrhosis (METAVIR score F4)

*Note:* AATD‐LD, AATD‐associated liver disease; BMI, body mass index; METAVIR, meta‐analysis of histological data in viral hepatitis; MRE, magnetic resonance elastography, VCTE, vibration‐controlled transient elastography.

**FIGURE 2:**
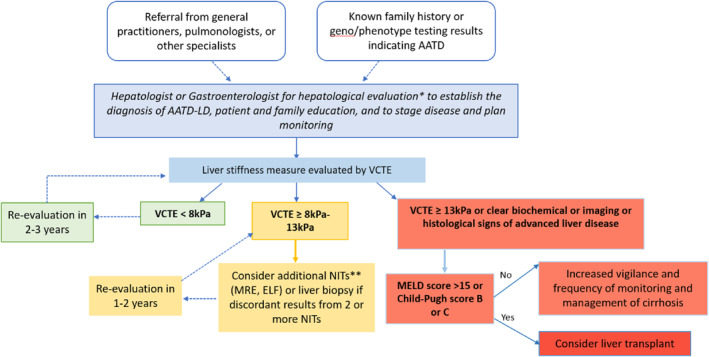
Proposed monitoring approach for alpha1‐antitrypsin deficiency‐associated liver disease (AATD‐LD). Individuals diagnosed with severe alpha1‐antitrypsin deficiency (AATD) should be referred for a hepatological evaluation to establish diagnosis of AATD‐LD, to stage disease, and plan monitoring. The first step is the evaluation of liver stiffness by vibration‐controlled transient elastography (VCTE). If advanced fibrosis is present, additional confirmatory testing is recommended. Monitoring intervals for the development of progressive AATD‐LD depend on baseline VCTE score and clinical symptoms. 1. Check for liver risk factors (metabolic syndrome, alcohol use disorder, ferritin, transferrin saturation, ceruloplasmin, auto‐immune liver disease screening, HBV, HCV, family history). 2. Test AST/ALT, GGT, ALP, FIB‐4, bilirubin, albumin, INR, and platelet count. *Note that the following may be performed by any clinician prior to hepatology referral for expediency. **NIT = non‐interventional tests.

The goal of the Delphi process was to build an approach for liver disease evaluation and risk stratification for individuals with AATD based on the presence of fibrosis. Early‐stage strong consensus was that a stepwise approach to monitoring AATD‐LD was appropriate. However, during the consensus building stage after the end of data collection, panelists agreed that this type of approach was not indicated for a rare disease, which was a change. Based on the consensus built using the Delphi process, the panel proposed an approach to monitoring Pi*ZZ patients for AATD‐LD, as shown in Figure 2. The recommendations include starting with a referral to a gastroenterologist or hepatologist for evaluation of underlying liver disease and fibrosis staging with VCTE. Evaluation of additional risk factors for liver disease was also determined to be an important part of the process. Confirmatory testing for advanced fibrosis with additional NITs if VCTE is ≥ 8 kPa as well as intervals for repeating VCTE measurements to monitor for development liver fibrosis are suggested. Because there are limited validated data to support the proposed threshold measurements, use of the proposed cutoffs represents a pragmatic approach to clinical care and a starting point for further validation studies.

## 5 Discussion

AATD is a rare genetic condition that can result in liver disease. Because it is not commonly encountered in clinical practice, there are limited data to inform the initial evaluation and monitoring of AATD‐LD [4, 9]. The present study obtained novel information from 20 clinician specialists from across the globe who care for individuals with AATD, including perspectives of pulmonologists, gastroenterologists, and hepatologists. Important data on key variables, rankings and evaluation of NITs, use of ultrasound and liver biopsy, and relevant thresholds for NITs to determine level of progression and vigilance of monitoring patients with AATD‐LD were explored using the Delphi method.

A key finding of the study is that most clinicians assess Pi*ZZ patients holistically. The contributions of comorbidities and lifestyle are also considered when formulating impressions for fibrosis risk and progression of liver disease. These considerations include the presence of other comorbid liver diseases or metabolic risk factors such as diabetes, obesity and alcohol use. Further studies are needed to define how these factors should influence the frequency of monitoring. Notably, in a recent longitudinal study, obese individuals as well as subjects with liver steatosis seem to progress more often [22].

It was difficult for the clinician panelists to provide definitive cutoffs for thresholds for NITs that correspond to progressive fibrosis categorical stages. Instead, fibrosis progression was characterized on a continuous spectrum. Ultimately, a strong consensus was reached around the VCTE thresholds, indicating > 80% of panelists endorsed the suggested cut points, with an emphasis that the threshold may be associated with a given METAVIR fibrosis score.

The recommendations for use of VCTE in fibrosis as a first assessment for AATD‐LD are consistent with the known performance characteristics of VCTE. VCTE seems to outperform other methods for detection of advanced fibrosis and for prediction of development of future hepatic end‐points but is less accurate in lower fibrosis stages [7, 19, 22]. The EASL clinical practice guidelines on genetic cholestatic liver diseases were published after the conduct of the study, and were not available to the panelist at the time. These guidelines are the first to address the use of NITs in AATD‐LD fibrosis staging and support the recommendation for VCTE as a method to estimate liver fibrosis [9]. Notably, the consensus of the Delphi panel for a VCTE cut‐off of < 8 kPa as the threshold for minimal fibrosis (F0‐1) is different from AATD‐LD specific cutoffs previously reported and will require further validation [7, 19, 23]. The cutoff of < 8 kPa to stratify as low risk is recommended in European guidelines published at the time of the study as well as recent US guidelines [15, 16, 17]. This suggests panelist may favor a single cut point that is not disease specific, which may be the most pragmatic approach when data and clinician experience in AATD‐LD are limited. The suggested threshold for cirrhosis is in line with some but not all guidelines, and almost no AATD‐LD‐specific data exist [24, 25]. The present Delphi panel also proposed an approach to monitoring Pi*ZZ patients for AATD‐LD. Since the data on liver disease progression in AATD‐LD are very limited [22, 26], the recommendations are likely based on guidelines from other liver disorders. Notably, subjects with AATD‐LD‐related liver cirrhosis were suggested to decompensate faster than individuals without AAT mutations [27, 28], so closer monitoring may be warranted, even though data on severe AATD genotypes such as Pi*ZZ are scarce [29].

The Delphi method provides a unique opportunity to address the study aim by soliciting real‐world knowledge from expert clinicians who care for AATD patients that is used to develop reasonable consensus statements for the broader community. The panel was able to build consensus while avoiding direct confrontation. Anonymity and confidentiality were ensured during the structured process to encourage panelists to reassess initial judgments in light of what colleagues had to say [30]. Blinding panelist identities helped prevent bias that could occur from name recognition and level of experience in the field. The Delphi method also has some limitations. Efforts were made to identify and include thought leaders from geographically diverse locations, but the small sample size may limit full generalizability as the participants were from the US, UK, and Europe without representation from Australia or Asia. Clinician panelists who participated may not reflect the full community of AATD clinicians, and the study cannot measure or control this potential bias, including those experts who chose not to participate. The responses of clinician panelists reflect unique patient populations and approaches to monitoring fibrosis progression with NITs. However, given the rarity of AATD‐LD, consensus statements from this study are less likely to encounter disagreement in clinical practice.

The study has several limitations that should be acknowledged. First, a non‐response analysis was not performed to investigate whether the answers from these potential panelists would skew the results. Second, the panelists had difficulty in providing specific numeric values for other NIT thresholds (MRE, APRI, FIB‐4) given the lack of evidence and absence of robust validation of NIT measurements with corresponding liver biopsy in a rare disease population. While MRE is considered a particularly accurate NIT for liver fibrosis for many conditions, data specific for AATD‐LD are limited [16, 23, 31]. As a result, it is not a surprise that a consensus around meaningful threshold values for MRE was not established. More surprisingly, consensus around the common blood‐based NITs (APRI and FIB‐4) were also not established despite robust use of these in other liver conditions and relatively disease agnostic cut‐off points. This may reflect a lack of experience in use with this rare disease population and more comfort in the reliability of VCTE as a liver stiffness measure that is reproducible. Pragmatically, it could also be explained by the change in consensus from the first round that fibrosis assessment should include a stepwise approach to the final consensus that fibrosis assessment should start with LSM by VCTE. Another potential limitation is that the questionnaire did not include questions to elicit specific information around the use of controlled attenuation parameter (CAP) scores as a measurement of steatosis, which is known to be a feature on liver biopsy in AATD as well as on VCTE [7, 8]. The presence of steatosis is not a risk factor for baseline fibrosis on biopsy in AATD [7], but might be associated with faster fibrosis progression. Its effect on VCTE accuracy has not been evaluated previously and needs to be addressed in future studies [22].

In conclusion, the need to generate evidence for disease‐specific and clinically important thresholds for NITs in the AATD‐LD population is clear. The results of this Delphi panel study address areas missing from previous clinical AATD guidelines due to the small number of studies on this rare liver disease. Most individuals with AATD are not cared for by a specialist with expertise in this rare disease and an useful, widely applicable approach is needed. The proposed approach for fibrosis staging and monitoring is based on the best available evidence and expert consensus opinion. The final consensus statements that emerged addressed the use of common clinical tools in AATD‐LD, including elastography, ultrasound, and liver biopsy, in a manner that is specific and actionable for any clinician. In future studies, it will be important to validate this approach and incorporate the patients' perspective in future recommendations. Once established, these will provide relevant and important information for patient care and be useful to determine guidance for treatment pathways in the advent of pharmacological therapies for AATD‐LD [11, 32].

## Author Contributions

M.A.P. and J.R. analyzed the data. V.C.C. and P.S. drafted, reviewed and edited the manuscript. All authors conceived and designed the study, interpreted the data, and read and approved the final version of the manuscript.

## Conflicts of Interest

V.C. has received grant support from Arrowhead Pharmaceuticals, Vertex Pharmaceuticals, Novo Nordisk, Takeda Pharmaceuticals, and Hamni Pharmaceuticals. Consulting fees were received from Takeda Pharmaceuticals and BioMarin Pharmaceuticals. M.P. and J.R. are full‐time employees of RTI Health Solutions, an independent nonprofit research organization, which was retained by Takeda to conduct the research which is the subject of this manuscript. Their compensation is unconnected to the studies on which they work on. R.L. serves as a consultant to Aardvark Therapeutics, Altimmune, Arrowhead Pharmaceuticals, AstraZeneca, Cascade Pharmaceuticals, Eli Lilly, Gilead, Glympse bio, Inipharma, Intercept, Inventiva, Ionis, Janssen Inc., Lipidio, Madrigal, Neurobo, Novo Nordisk, Merck, Pfizer, Sagimet, 89 bio, Takeda, Terns Pharmaceuticals and Viking Therapeutics. R.L. has stock options in Sagimet biosciences. In addition, his institution received research grants from Arrowhead Pharmaceuticals, Astrazeneca, Boehringer‐Ingelheim, Bristol‐Myers Squibb, Eli Lilly, Galectin Therapeutics, Gilead, Intercept, Hanmi, Intercept, Inventiva, Ionis, Janssen, Madrigal Pharmaceuticals, Merck, Novo Nordisk, Pfizer, Sonic Incytes and Terns Pharmaceuticals. Co‐founder of LipoNexus Inc. A.M.T. has received grant support or honoraria from CSL Behring, Grifols Inc., Vertex Pharmaceuticals, AstraZeneca, GSK, Chiesi and Takeda Pharmaceuticals. P.S. reports receiving grants and honoraria from Arrowhead Pharmaceuticals, CSL Behring, Grifols Inc., consulting fees or honoraria from AIRNA, Alnylam Pharmaceuticals, Arrowhead Pharmaceuticals, BioMarin Pharmaceutical, BridgeBio, Dicerna Pharmaceuticals, GSK, Ipsen, Intellia Pharmaceuticals, Takeda Pharmaceuticals, Novo Nordisk and Ono Pharmaceuticals, participating in leadership or fiduciary roles in Alpha1‐Deutschland, Alpha1 Global, and material transfer support for Vertex Pharmaceuticals and Dicerna Pharmaceuticals.

## Supporting information

Supporting Information S1

Figure S2

Figure S3

Figure S4

Figure S5

Figure S6

Figure S7

Figure S8

Figure S9

Figure S10

Figure S11

## Data Availability

The data that support the findings of this study are available from the corresponding author upon reasonable request.
