# Supplementary material for: Diagnosis and Monitoring Pathways Using Non‐Invasive Tests in Patients With Alpha‐1 Antitrypsin Deficiency‐Associated Liver Disease: Results From an Expert Delphi Panel
Source: United European Gastroenterol J. 2025 Mar 12;13(8):1446–55. doi: 10.1002/ueg2.70009 (PMC12529016; doi:10.1002/ueg2.70009)
Supplement: Supplementary file 1 — Supporting Information S1 [file UEG2-13-1446-s009.docx]

**Supplemental material to:**

**Diagnosis and monitoring pathways using non-invasive tests in patients with alpha-1 antitrypsin deficiency-associated liver disease: results from an expert Delphi panel**

Virginia C. Clark,^1^ Mark A. Price,^2^ Jon Russo,^2^ Rohit Loomba,^3^ Alice M Turner,^4^ Pavel Strnad^5^

**Supplemental Figure 1. Study Design Flow Chart**


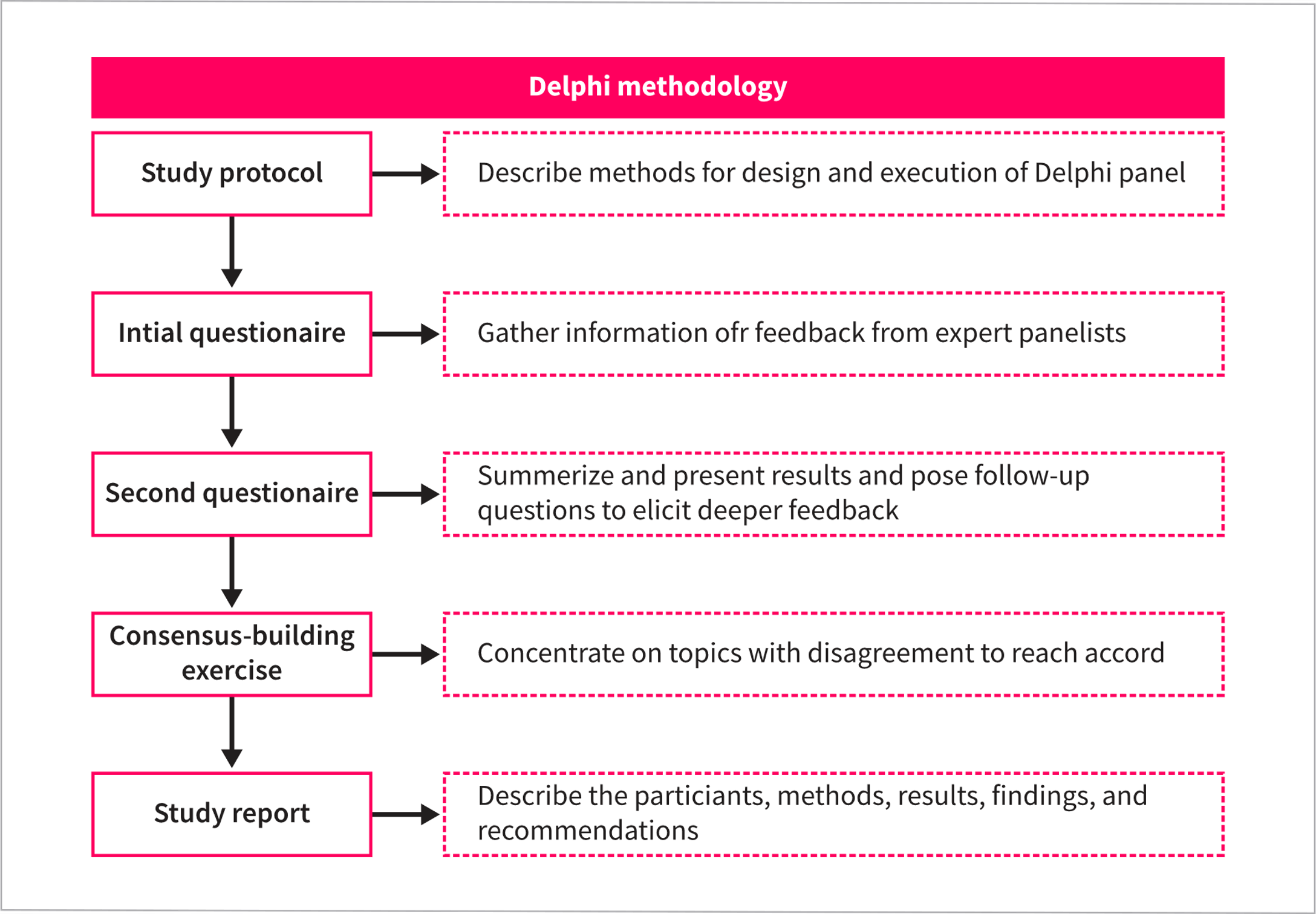


**Supplemental Table 1. Data Collection Process**

|  | **Delphi questionnaire 1** | **Delphi questionnaire 2** | **Consensus-building exercises** |
| --- | --- | --- | --- |
| Informed consent | X |  |  |
| Inclusion/exclusion criteria | X |  |  |
| Demographics (specialty, years in practice, education, approximate number of patients with AATD-LD followed yearly)^a^ | X |  |  |
| Initial data collection on similarities and variations in monitoring and noninvasive assessment pathways and thresholds | X |  |  |
| Presentation of initial questionnaire results and a deeper examination of areas of disagreement |  | X |  |
| Final discussion and derivation of consensus statements |  |  | X |

AATD-LD, AATD-associated liver disease

**Supplemental Table 2. Consensus statements from Delphi Round 1**

| Statement | Panel Consensus | | | |
| --- | --- | --- | --- | --- |
|  | **Complete**  **(100%)** | **Strong**  **(80-99%)** | **Moderate**  **(60-79%)** | **Weak**  **(50-59%)** |
| VCTE is used for diagnosis and/or monitoring for patients with AATD-LD | X |  |  |  |
| VCTE and other sophisticated elastography or liver stiffness imaging techniques such as MRE are considered the most informative NIT in monitoring fibrosis progression in AATD-LD | X |  |  |  |
| Patients with AATD-LD are clinically assessed using a combination of elastography, biochemical tests, and ultrasound | X |  |  |  |
| Patients with AATD-LD are assessed via VCTE every 2-3 years if no additional risk factors for liver disease are present | X |  |  |  |
| A key determinant of NIT use is resource availability. Rural and smaller practice settings rely more on serum-based NITs in the absence of elastography | X |  |  |  |
| Patients with AATD-LD are assessed via biochemical tests every year to monitor fibrosis progression |  | X |  |  |
| NITs not used for diagnosis or monitoring include ARFI, 2D-shear wave elastography, NAFLD fibrosis score, FibroTest/FibroSure, and Hepascore | X |  |  |  |
| ELF test is generally not used for the diagnosis and/or monitoring for AATD-LD |  |  | X |  |

AATD-LD, AATD-associated liver disease; ARFI, acoustic radiation force impulse; ELF test, enhanced liver fibrosis test; MRE, magnetic resonance elastography, NIT, non-invasive test; VCTE, vibration-controlled transient elastography

**Supplemental Table 3. Consensus Statements from Delphi Round 2**

| Statement | Panel Consensus | | | |
| --- | --- | --- | --- | --- |
|  | **Complete**  **(100%)** | **Strong**  **(80-99%)** | **Moderate**  **(60-79%)** | **Weak**  **(50-59%)** |
| A clear classification system of AATD-LD progression is needed as well as a stepwise approach to evaluation of AATD-LD | X |  |  |  |
| The most common clinical approach to initial evaluation of a patient with AATD-LD is referral to a hepatologist and use of VCTE, laboratory measures, ultrasound, and an option clinical evaluation | X |  |  |  |
| Frequency of evaluation of patients with AATD-LD is based on METAVIR score with lower scores evaluated every 2-3 years and higher scores evaluated every 6-12 months | X |  |  |  |
| VCTE is the most used NIT for the first evaluation of a patient with AATD-LD | X |  |  |  |
| Elastography (VCTE, MRE) was ranked as most valuable NIT to monitor AATD-LD progression by 70% of panelists and still highly valuable by the remaining panelists |  | X |  |  |
| Limited use of MRE as a diagnostic and/or monitoring tool is due to cost and lack of availability |  | X |  |  |
| Use of additional NITs to monitor AATD-LD progression is lacking | X |  |  |  |
| Validated data on FIB-4 values in the AATD-LD population and its value in monitoring disease progression is lacking | X |  |  |  |

AATD-LD, AATD-associated liver disease; FIB-4, fibrosis 4 index; METAVIR, meta-analysis of histological data in viral hepatitis; MRE, magnetic resonance elastography, NIT, non-invasive test; VCTE, vibration-controlled transient elastography

**Supplemental Table 4. Results of Consensus Building Exercises**

| **Topic explored by Delphi panel** | **Findings endorsed by panelists** | **Supporting quotes from Delphi panelists** |
| --- | --- | --- |
| **Ranking of NITs** |  |  |
| Elastography | **Complete** consensus was reached among panelists that elastography (e.g., VCTE; MRE) is the most or second-most valuable noninvasive monitoring approach to evaluate AATD-LD progression | “VCTE is well-implemented as expected for a gold standard NIT. MRE is not available in Europe. From a payer’s perspective I do not see a reason to reimburse MRE at this point.” |
| Value of other NITs | Some panelists indicated the context where other NITs can provide value | “If ELF is in line with LSM by VCTE, it increases my confidence in my risk assessment.”    “Discordance is common but a concordant FIB-4 for < 1.3 (not advanced fibrosis) or > 2.67 (advanced fibrosis) would increase confidence.” |
| **Evaluation of NITs** |  |  |
| Diagnosis and monitoring | **Complete** consensus was reached with all clinician panelists unanimously agreeing that diagnosis and monitoring need to have their own separate approaches.  Some panelists indicated that with the shortage of hepatologists and delays for consultations they appreciate when another specialist (e.g., general practitioner or pulmonologist) orders tests for AST/ALT, GGT, ALP, and platelet count, checks for liver risk factors (metabolic syndrome, alcohol use disorder, HBV, HCV, familial history), or even orders ultrasound and elastography if they are able. | “Diagnosis and monitoring need two different approaches.”    “I think if there is a delay getting to see the hepatologist then VCTE, biochemical testing including viral [hepatitis screening], and AFP and US liver can all be helpful to have already reported at specialist assessment.”    “[It is] helpful if a patient presents with ultrasounds and with lab tests. Then [I would] perform a Fibroscan. This should be the pre-hepatology workup. Interpreting tests requires some experience [from the hepatologist]. |
| Use of traditional ultrasound in AATD-LD | Clinician panelists indicated **strong** consensus that ultrasound has value in an initial evaluation to collect baseline information on liver morphology and to evaluate the biliary tree, portal vein, and identify potential steatosis and/or parenchymal masses and nodules. However, ultrasound is less valuable for initial staging and ongoing monitoring as a single method.  Repeat ultrasounds in patients with AATD-LD are used for screening for hepatoma, signs of liver decompensation, and hepatocellular carcinoma. | “Decision to use ultrasound depends on stage not age.”    “I think a baseline ultrasound is OK, then only in the course of HCC screening [in advanced disease].” |
| **Use of biopsy** |  |  |
| Biopsy use | Clinician panelists reached **complete** consensus and unanimously agreed that biopsy **may be considered** in the following circumstances in patients with AATD-LD:   - To exclude competing causes of liver injury and chronic liver diseases - Elevated liver enzymes - Significant fibrosis is detected on VCTE - To confirm significant liver disease - To confirm in the presence of discordant NIT results | “The critical word to address is ‘can,’ is that what is recommended?”    “The value of biopsy is where there is uncertainty” |
| **Risk stratification and NIT thresholds** | | |
| Evaluation frequency | Clinician panelists reached **complete** consensus and unanimously agreed that less frequent evaluations occur with lower METAVIR scores (e.g., F0 and F1) on the order of every few years (2-3 years), while more frequent evaluations occur with higher METAVIR scores (typically every 6 months-1 year). Evaluation schedules also vary among presence of different comorbidities and BMI. | “[for] Alpha 1 with low degrees of fibrosis, 2-5 years. [I] would be reluctant to monitor with great frequency.” |
| Risk stratification when pharmacological treatment becomes available | Clinician panelists reached **moderate** consensus that knowing when a patient has progressed to F2 is important because it is an indication of liver injury, and that monitoring vigilance should be increased.  Clinician panelists agreed that the distinction between F0 vs. F1 and F2 vs. F3 are not clinically meaningful. |  |
| Elastography | Clinician panelists reached **strong** consensus that a LSM value of 8 kPa or greater denotes the progression stage that heightened vigilance should occur with monitoring. With a few exceptions and with a bit of overlap, panelists generally indicated that typical VCTE kPa values for patients with AATD-LD that are associated with fibrosis scores could be as follows:   - F2 = 8 kPa or greater - F3 = 10 kPa or greater - F4 = 13 kPa or greater   While some panelists expressed the difficulty of defining clear, mutually exclusive ranges, they agreed that vigilance should be exercised as patients yield LSM values around these 3 key thresholds so that adjustments could be made regarding monitoring frequency and techniques and eventual treatment decisions. |  |
| MRE | Panelists were unable to assign specific MRE values for each fibrosis score but 1 panelist suggested the following:   - F2 = 3.3 kPa or greater - F3 = 3.6 kPa or greater - F4 = 4.67 kPa or greater |  |
| FIB-4 | Panelists were unable to assign specific FIB-4 values for each fibrosis score but generally agreed that a score of 3 or more indicates severe disease (F4) |  |
| APRI | Panelists were not confident to provide APRI values for each fibrosis score |  |

AATD-LD, AATD-associated liver disease; AFP, alpha-fetoprotein; APRI, aspartate aminotransferase-to-platelet-ratio index; ALP, alkaline phosphatase; ALT, alanine aminotransferase; AST, aspartate aminotransferase; BMI, body mass index; F, fibrosis stage; FIB-4, fibrosis 4 index; GGT, gamma-glutamyl transferase; HBV, hepatitis B virus; HCC, hepatocellular carcinoma; HCV, hepatitis C virus; LSM, liver stiffness measurement; METAVIR, meta-analysis of histological data in viral hepatitis; MRE, magnetic resonance elastography, NIT, non-invasive test; US, ultrasound; VCTE, vibration-controlled transient elastography
